# Biliary Cystadenoma Mimicking Hydatid Cyst

**DOI:** 10.4103/1319-3767.54744

**Published:** 2009-07

**Authors:** M. H. Raza, A. Z. Rab, Shehtaj Khan, Reyaz Ahmad

**Affiliations:** Department of Surgery, J.N. Medical College, AMU, Aligarh, U.P, India. E-mail: shehtaj@hotmail.com

Sir,

Biliary cystadenomas are rare hepatobiliary cystic tumors, more common in women. We report one such tumor that presented with obstructive jaundice and mimicked a hydatid cyst of the common bile duct (CBD). Cystadenoma was diagnosed only after biopsy but, due to wide excision, there was no residual or recurrent tumor.

Biliary cystadenomas are rare cystic tumors that arise in the liver or less frequently in the extrahepatic biliary system. They are more common in middle-aged women, their most favored site being the right hepatic lobe. It is extremely rare for an intrahepatic cystadenoma to extend into the CBD. Less than 10 cases have been reported in the medical literature.[1]

Presentation is frequently nonspecific: Patients may complain of abdominal pain, anorexia and nausea. Liver function tests are frequently abnormal and extrahepatic lesions tend to present with obstructive jaundice.

A 20-year-old female was admitted with a 10-day history of jaundice and a 3-day history of increasing right upper quadrant colicky pain. She had also experienced previous intermittent episodes of jaundice. Liver function tests were abnormal on admission, with a serum bilirubin of 4.8 mg/dL and alkaline phosphatase of 22 KAU. An ultrasound scan revealed multiple coiled ascaris in the CBD, seen as bunches and also appearing calcified. Intrahepatic ducts and CBD were dilatated (16 mm). Periportal lymphadenopathy was present.

**Figure 1:**
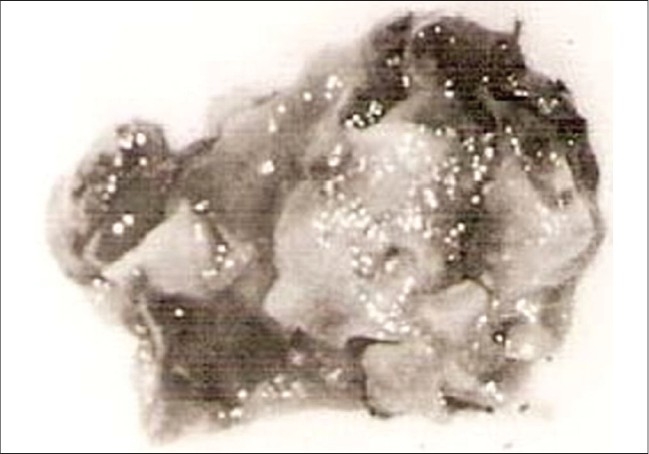
Excised cystadenoma

There was a diagnostic dilemma with which an exploration was undertaken. At laparotomy, there were multiple cysts filling up the CBD and a perop diagnosis of hydatid disease was made. Excision of the cyst [Figure 1] with removal of an enlarged regional lymph node was performed and a T-tube was left *in situ*.

Histology showed the tissue to be cystadenoma. The patient had a smooth postoperative course with T-tube removal performed on the 14^th^ postop day. She was discharged and closely followed-up.

Biliary cystadenomas are rare and their similarity to hydatid disease can make them difficult to diagnose. The radiological appearance of biliary cystadenomas is quite specific. Ultrasound may show an anechogenic mass with internal septations that are highly echogenic, which, in our case, led us to think of it as calcified ascaris. Computed tomography helps with the finding of a smooth thick-walled cyst with fine internal septae,[2] but it could not be performed in this case due to financial constraints.

At laparotomy, these lesions can be difficult to localize as they are usually soft, difficult to palpate and pose minimal or no resistance to biliary probes. Therefore, the use of intraoperative choledochoscopy and cholangiography[3] has been advocated.

In 1892, the first case of successful resection for a biliary cystadenoma was reported.[4] Since this time several treatment strategies have been suggested: Simple enucleation, local excision either with a cuff of bile duct or liver and radical excision, including hemihepatectomy if appropriate. However, these tumors have a low malignant potential and unless excised completely, tend to reoccur. The largest reported series of 15 cases is by Lewis *et al.*, who concluded that while formal lobar resection is frequently carried out, local complete excision is just as effective provided the neoplasm is entirely removed and is associated with low rates of morbidity.[5]

In developing countries, a wide local excision with regular follow-up would be more appropriate considering the limited availability of adequate back up for a more radical procedure at most of the centers.
